# Electrospun cobalt-doped 2D-MoSe_2_/polypyrrole hybrid-based carbon nanofibers as electrochemical sensing platforms

**DOI:** 10.1007/s00604-023-06078-2

**Published:** 2024-01-04

**Authors:** Gamze Celik Cogal, Sadik Cogal, Peter Machata, Aysegul Uygun Oksuz, Maria Omastová

**Affiliations:** 1 Polymer Institute, Slovak Academy of Sciences, Dubravska cesta 9, 84541 Bratislava, Slovakia; 2 Faculty of Arts and Science, Department of Chemistry, Suleyman Demirel University, 32000 Isparta, Türkiye; 3 Faculty of Arts and Science, Department of Chemistry, Burdur Mehmet Akif Ersoy University, 15030 Burdur, Türkiye

**Keywords:** Molybdenum diselenide, Carbon nanofibers, Electrospinning, Electrochemical sensor, Cyclic voltammetry, Differential pulse voltammetry, Dopamine, Ascorbic acid, Uric acid

## Abstract

A novel cobalt-doped two-dimensional molybdenum diselenide/polypyrrole hybrid-based carbon nanofiber (Co/MoSe_2_/PPy@CNF) was prepared using the hydrothermal method followed by electrospinning technique. The structural and morphological properties of the 2D-TMD@CNF-based hybrids were characterized through X-ray photoelectron spectroscopy (XPS), scanning electron microscopy (SEM), energy-dispersive spectrometry (EDS), and transmission electron microscopy (TEM). The Co-MoSe_2_/PPy@CNF exhibited large surface area, porous structure, and improved active sites due to the synergistic effect of the components. The electrochemical and electrocatalytic characteristics of the 2D-TMD@CNF-modified electrodes were also investigated using cyclic voltammetry (CV) and differential pulse voltammetry (DPV) techniques. The Co/MoSe_2_/PPy@CNF electrode was used as an electrochemical sensor for simultaneous detection of ascorbic acid (AA), dopamine (DA), and uric acid (UA) and showed enhanced catalytic activity and sensitivity. Using DPV measurements, the Co/MoSe_2_/PPy@CNF demonstrated wide linear ranges of 30–3212 μM for AA, 1.2–536 μM for DA, and 10–1071 μM for UA with low detection limits of 6.32, 0.45, and 0.81 μM, respectively. The developed sensor with the Co/MoSe_2_/PPy@CNF-modified electrode was also applied to a human urine sample and gave recoveries ranging from 94.0 to 105.5% (*n* = 3) for AA, DA, and UA. Furthermore, the Co/MoSe_2_/PPy@CNF-based sensor exhibited good selectivity and reproducibility for the detection of AA, DA, and UA.

**Graphical abstract:**

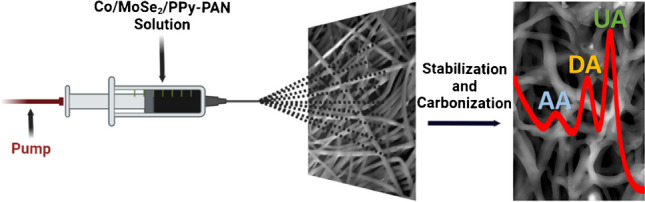

**Supplementary Information:**

The online version contains supplementary material available at 10.1007/s00604-023-06078-2.

## Introduction

The human body is an intricately balanced system containing countless molecules and compounds that work together to maintain optimal health and function. These molecules include dopamine (DA), ascorbic acid (AA), and uric acid (UA), which have different structural and functional properties and which play key roles in the physiological well-being of the organism [1]. Recognized as the “euphoria-inducing” neurotransmitter, DA is a key neurotransmitter that functions as a chemical messenger with central involvement in the central nervous system. AA, scientifically identified as L-ascorbic acid and recognized as vitamin C, is a water-soluble essential micronutrient that plays a central role in a wide range of biochemical pathways essential to human physiology [2]. UA exhibits antioxidant properties by effectively scavenging reactive oxygen species, thereby protecting cellular components from oxidative stress-induced damage [3]. These molecules have similar structures and tend to coexist in biological samples, leading to potential interference in their respective analyses. Therefore, it is critical for analytical and diagnostic applications to be able to detect these molecules simultaneously, efficiently, reliably, selectively, and rapidly [4, 5]. Various techniques, including ion chromatography, electrophoresis, calorimetry, and chemiluminescence, have been used to analyze these molecules, but they require complex equipment, trained person, and long time to complete an analysis [6]. Compared with these techniques, electrochemical sensors offer several advantages such as ease of use, affordability, rapid response, increased sensitivity, and excellent selectivity [7, 8]. AA, DA, and UA are electroactive molecules and can therefore be determined by electrochemical methods [9]. Electrochemical sensors built with nanomaterials have found widespread use across multiple application fields due to their analytical advantages, such as their simplicity, remarkable sensitivity, high selectivity, cost-effectiveness, and powerful response signals [10–14]. For this reason, enormous research efforts have been directed toward the enhancement of novel electrodes made from diverse materials. Nanomaterials with various benefits, including improved structural features (i.e., substantial surface area, high porosity), excellent electrical conductivity, high biocompatibility, and fine particle size, have been selected to augment the sensitivity and detection limit of electrochemical sensors [15]. Among various materials, two-dimensional (2D) transition metal dichalcogenides (TMDs), which are analogous to graphene, have garnered significant attention in electrochemical sensing applications owing to their layered structure, extensive surface area, high chemical stability, and excellent adsorption capacity [16]. 2D-TMD materials contain edge sites, which enable the materials to show high catalytic activities, and their catalytic performance can be controlled by controlling the number of layers [17]. In particular, molybdenum diselenide (MoSe_2_), as a typical TMD material, has become very popular for electrochemical applications, including sensors, electrocatalysis, and energy storage due to its unique properties [18–20]. MoSe_2_ and its composites have attracted widespread attention due to their unique properties, such as low cost, high electrocatalytic abilities, natural abundance, good electrochemical stability, and band gap [21, 22]. However, bulk pristine TMD materials, such as MoSe_2_, suffer from low electric conductivity, agglomeration, and a rapid decrease in capacitive properties, which limit their electrocatalytic performance [22, 23]. Therefore, various approaches have been reported to prepare TMD materials with controlled and enhanced catalytic properties [24]. It has been reported that elemental doping with nonnoble metals (Co, Ni, Fe, etc.) can improve the electronic conductivity and electron density of MoSe_2_ for electrochemical sensors and facilitate charge transfer properties during redox reactions [25–27]. Furthermore, conductive polymers (CPs) can be utilized as a conductive template with a large surface area to preserve more TMD nanolayers, leading to the formation of more active sites and improving their electrocatalytic properties [28]. Recently, it has been reported that the combination of MoSe_2_, elemental doping, and conducting polymer results in improved electrochemical performance for electrocatalytic performance [29]. In addition to these methods, the preparation of TMD-based nanofibers can further increase their catalytic performances for various electrochemical applications [30, 31].

Among conventional fiber production methods, electrospinning is a highly efficient technology with many advantages; as fiber morphologies can be controlled, finer fibers can be produced and increasingly compatible materials can be fabricated [32]. Although new approaches have been developed in this field of technology since the 20th century, there has been increasing interest in terms of simple production techniques, ease of setup, application areas, and possible future biomedical applications [33]. Therefore, among various nanomaterials, electrospun nanofibers (NFs) have been widely used in the field of electrochemical sensors due to their large surface area, good surface modifications, porous structure, and high biocompatibility [34, 35]. In addition, carbon nanofibers (CNFs) are a perfect support for MoSe_2_ to eliminate the limitations mentioned above. CNFs, which have a diameter of 10–500 nm and a length up to 10 μm, can be produced by electrospinning followed by a carbonization process. CNFs exhibit a large surface area, high electron transfer potential, and excellent mechanical structure due to the presence of more edges on their outer walls [36, 37]. Much focus has been placed on the combination of highly active MoSe_2_ with carbonaceous materials for preparing functional materials with enhanced electrical and structural properties for electrochemical applications [38]. Here, we report the production of 2D-TMD-based CNFs and their application as electrochemical sensors for the detection of AA, DA, and UA. A hydrothermal procedure was first used to prepare all 2D-TMD-based materials (MoSe_2_, MoSe_2_/PPy, Co/MoSe_2_, Co/MoSe_2_/PPy). Then, NFs and CNFs were fabricated by electrospinning and carbonization processes, respectively. The morphology and structure of 2D-TMD NFs and CNFs were characterized, and their electrochemical sensing features were investigated via different electrochemical techniques. The sensor has been effectively utilized for quantification in urine samples, demonstrating satisfactory recovery rates.

## Experimental

### Materials

Sodium molybdate dehydrate (Na_2_MoO_4_·2H_2_O) (Aldrich, >99%), polyacrylonitrile (PAN, 99%, Mw: 150,000 g/mol) (Sigma Aldrich, USA), N,N-dimethylformamide (DMF) (Sigma Aldrich, USA), selenium (Se) powder (Aldrich, 99.9%), potassium ferricyanide(III) [K_3_Fe(CN)_6_] (Sigma-Aldrich, 99 %), hydrazine hydrate (N_2_H_4_) (Sigma Aldrich, 50–60%), pyrrole (PPy) (Fluka, >99%), cobalt (II) nitrate hexahydrate [Co(NO_3_)_2_·6H_2_O] (Merck, 98%), HCl (Sigma Aldrich, 37%), sodium phosphate monobasic dihydrate (NaH_2_PO_4_∙2H_2_O) (Sigma Aldrich), sodium phosphate dibasic dihydrate (Na_2_HPO_4_∙2H_2_O) (Sigma Aldrich), and iron(III) chloride (FeCl_3_) (CentralChem, >99.9%) were commercially supplied and used as received.

### Synthesis of MoSe_2_ nanosheets

**Fig. 1:**
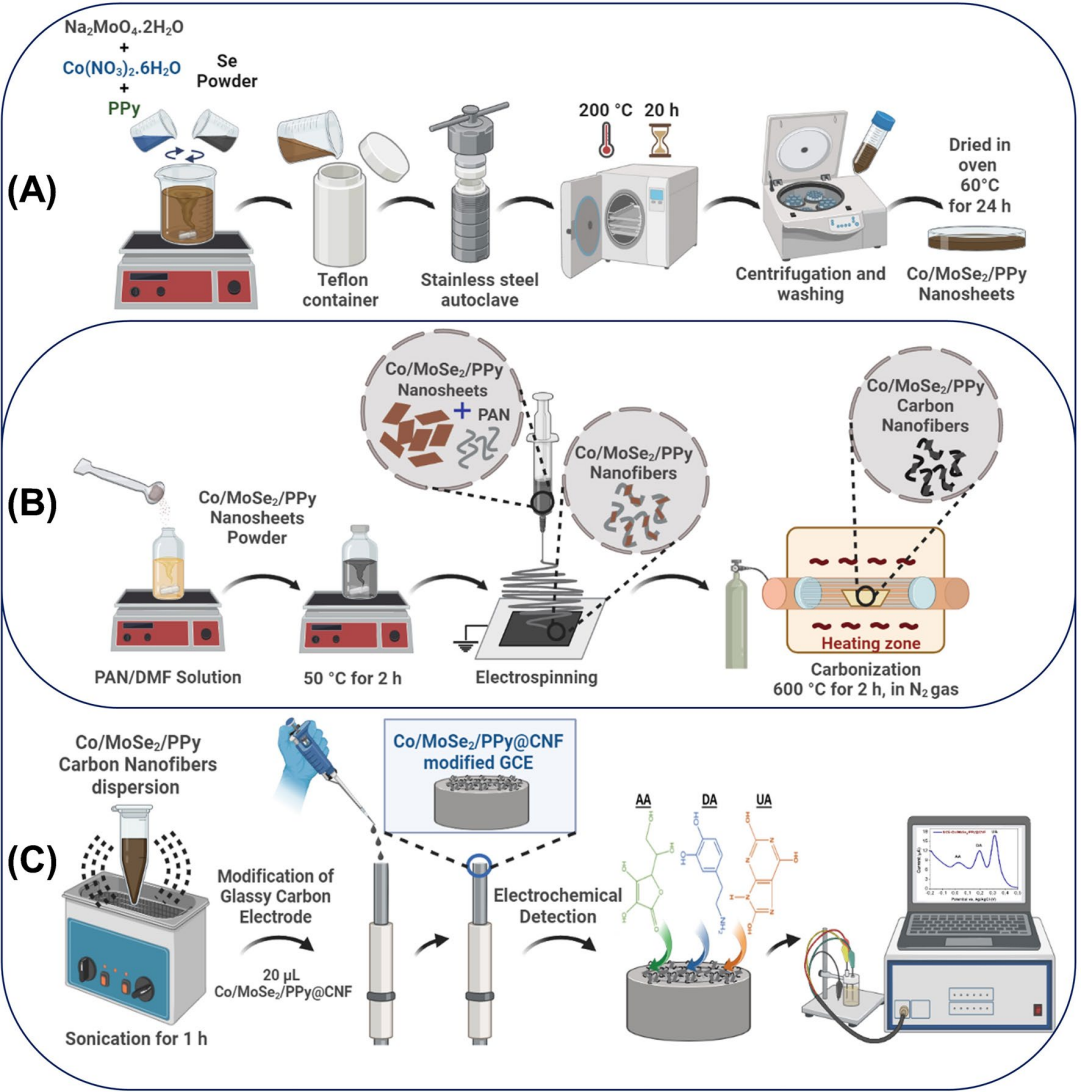
Schematic description of **A** synthetic hydrothermal procedure, **B** preparation of carbon nanofibers by electrospinning, and **C** fabrication of electrode and electrochemical detection

A simple and cost-effective hydrothermal technique was utilized to synthesize MoSe_2_ nanosheets. Therefore, 2 mmol of Na_2_MoO_4_·2H_2_O was dissolved in 40 mL of deionized (DI) water and then subjected to magnetic stirring (500 rpm) for approximately 1 h. In another flask, 4 mmol Se powder was dissolved in a 20 mL N_2_H_4_ (50–60%) solution and stirred at 500 rpm for 1 h. After homogeneous solutions were obtained, two solutions were mixed and stirred (500 rpm) for another 0.5 h to obtain a homogeneous reaction mixture. In the next step, the homogeneous mixture was moved to a hydrothermal Teflon-lined stainless-steel autoclave. The autoclave was heated in a furnace at 200 °C for 20 h. After the reaction was completed and the autoclave was naturally cooled to room temperature, the product was centrifuged at 6000 rpm for 5 min, which was washed several times with excess DI water to remove unreacted precursors and side products. Then, the final product was dried in a vacuum oven at 60 °C for 24 h. This procedure is schematically presented in Fig. 1A.

### Synthesis of MoSe_2_/PPy hybrids

Firstly, polypyrrole (PPy) was synthesized via an oxidative chemical polymerization (OCP). In a typical OCP process, 7.1 g of FeCl_3_ powder was dispersed in 1 M HCl in an ice bath and stirred in an ice bath at 0–5 °C for 30 min to obtain an oxidant solution. Then, 6.1 mL of pyrrole monomer was added to the prepared solution and refluxed for 16 h at room temperature. After that, the obtained product was filtered and washed several times with methanol and acetone solutions. The final black product was dried at 60 °C overnight in an oven. The preparation of the MoSe_2_/PPy sample was carried out as described above for the synthesis of MoSe_2_ nanosheets, but 100 mg of ultrasonically dispersed PPy was added to the Mo-salt solution to prepare MoSe_2_/PPy.

### Synthesis of cobalt-doped MoSe_2_/PPy hybrids

The preparation of cobalt-doped MoSe_2_/PPy was carried out as described above for the preparation of MoSe_2_/PPy samples, but 0.2 mmol Co(NO_3_)_2_·6H_2_O was added to the Mo-salt/PPy solution to obtain Co/MoSe_2_/PPy. As a control, a Co/MoSe_2_ sample was also prepared in the same way without adding PPy.

### Preparation of 2D-TMD@NFs

As-synthesized MoSe_2_, MoSe_2_/PPy, Co/MoSe_2_/PPy, and Co/MoSe_2_ were used as precursor materials for the preparation of electrospun nanofibers. PAN was used as a polymer support for NF preparation. The precursor 2D-TMD material/PAN (mass ratio 2:50) NFs were fabricated via a facile and low-cost electrospinning technique. The PAN polymer was dispersed in 20 mL of DMF for 2 h at 500 rpm under 50 °C for thorough mixing. Then, 25% of MoSe_2_-based materials were added to the homogeneously dispersed PAN (10 wt%) polymer solution. These mixtures were stored in an ultrasonic bath for 10 min and magnetically stirred at 500 rpm for 2 h under 50 °C. Black precursor-based electrospun fibers were fabricated using a Spellman high-voltage power source (Spellman High Voltage Electronics Corporation, USA) and syringe pump (New Era Pump Systems, Inc., USA). The black MoSe_2_-based/PAN solution was taken into a 10 mL syringe integrated with a grounded needle with a 0.41 mm diameter and size of 21 G. Afterward, the working voltage, tip collector distance, and flow rate of the electrospinning were fixed at 14.5 kV, 17 cm, and 1.5 mL/min, respectively. A fiber network consisting of all materials was obtained on an aluminum foil used as a collector (Fig. 1B).

### Carbonization of 2D-TMD@NFs

For use as sensor electrodes, spun MoSe_2_-based composite fibers were exposed to heat treatments to carbonize the PAN. To obtain high-performance CNFs, the films underwent stabilization and carbonization in a tubular quartz furnace. The as-spun NFs were first annealed in air at 260 °C for oxidative stabilization for 2 h, followed by heating to 600 °C under continuous N_2_ flow at a rate of 5 °C min^−1^ for 2 h. After that, all samples were cooled down spontaneously in the presence of N_2_ through the oven (Fig. 1B).

### Characterization of 2D-TMD@NFs and 2D-TMD@CNFs

The morphologies of MoSe_2_, MoSe_2_/PPy, Co/MoSe_2_, and Co/MoSe_2_/PPy NFs and their CNFs were investigated by scanning electron microscopy (SEM) with a FEI Quanta FEG 250 Model (USA) and high-resolution transmission electron microscopy (HRTEM) with a JEOL-2100 operated at 200 kV. To determine the valence state of the elements of the NFs and CNFs, X-ray photoelectron spectroscopy (XPS) measurements were implemented by NEXSA-G2, monochromated high-performance XPS spectrometer (Thermo Fisher Scientific, UK) with a monochromatic Al Kα (1486.68 eV) X-ray source.

### Electrochemical characterization of 2D-TMD@CNFs

The electrochemical properties of the modified electrodes were determined by cyclic voltammetry (CV), differential pulse voltammetry (DPV), and electrochemical impedance spectroscopy (EIS). All electrochemical experiments were performed using an SP-200 electrochemical analyzer (BioLogic Inst., France) fitted with a conventional three-electrode configuration and a 5 mL cylindrical electrolyte cell. MoSe_2_@CNF-, MoSe_2_/PPy@CNF-, Co/MoSe_2_@CNF-, and Co/MoSe_2_/PPy@CNF-coated GCEs with 3-mm diameters were used as working electrodes. Additionally, Ag/AgCl (1 M KCl) and platinum wire were used as the reference electrode and counter electrode, respectively. For electrode modification, 4 mg of 2D-TMD-CNF was dispersed in 1 mL of DMF and sonicated for 1 h to form a homogenous dispersion. Then, 10 μL from this dispersion was drop-coated on the smooth GCE surface and naturally dried in air at room temperature. To verify the electrochemical behaviors of the modified electrodes, CV and EIS were recorded in 5.0 mM [Fe(CN6)]^3−/4−^ containing 0.1 M KCl solution. The CV measurements were obtained at a scan rate of 50 m Vs^−1^ in a potential range from 0.0 to 0.5 V. EIS was recorded in the range of 100 mHz to 100 kHz at a voltage of 0.25 V. Electrochemical studies to identify DA, AA, and UA were performed using the DPV method in a potential range from −0.2 to +0.5 V in 0.1 M PBS (pH = 7.0) solution. A schematic of the arrangement is shown in Fig. 1C.

## Results and discussion

### Characterization of 2D-TMD@CNF nanocomposites

2D-TMD@CNF materials were prepared and applied in three steps. The first step was fabricating the 2D-TMD nanosheets through a hydrothermal reaction, and the second step involved producing CNFs by electrospinning and heat treatment. The final step was preparing the 2D-TMD@CNF-based working electrode and determining the electrochemical sensor behaviors and responses to biological molecules (Fig. 1).

**Fig. 2:**
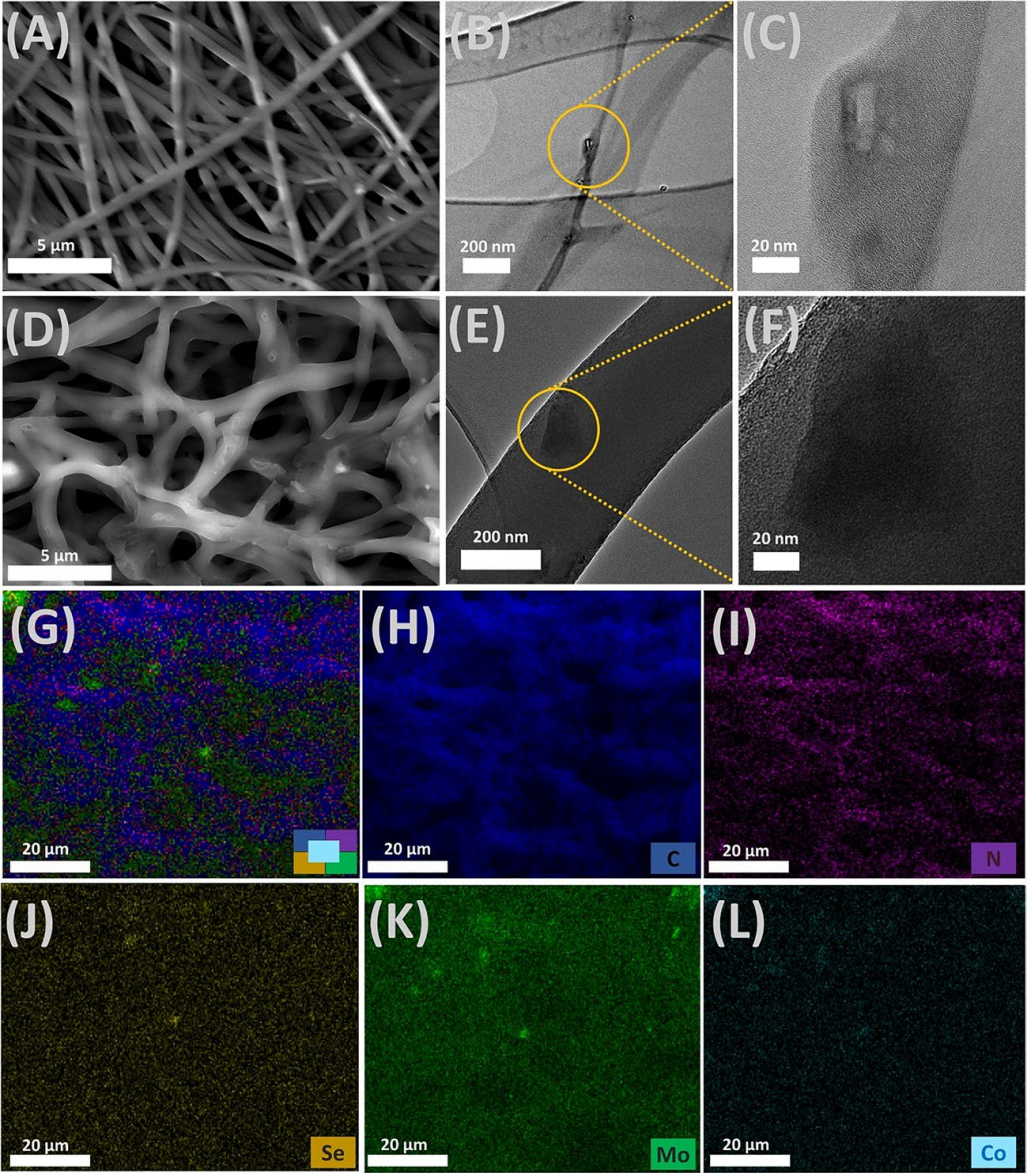
Characterizations of Co/MoSe_2_/PPy@NF and Co/MoSe_2_/PPy@CNF. **A** SEM images of Co/MoSe_2_/PPy@NF. **B**, **C** TEM image of Co/MoSe_2_/PPy@NF. **D** SEM images of Co/MoSe_2_/PPy@CNF. **E**, **F** TEM image of Co/MoSe_2_/PPy@CNF. **G**–**L** Corresponding elemental mappings for all elements, C, N, Se, Mo, and Co

SEM was performed to characterize the surface morphology and composition of the Co/MoSe_2_/PPy carbon nanofibers (Fig. 2). Figure 2A shows that Co/MoSe_2_/PPy@NF possessed a smooth surface morphology with diameters ranging from 395 to 469 nm. To determine the detailed structural properties, Co/MoSe_2_/PPy@NFs were further examined by transmission electron microscopy (TEM), as shown in Fig. 2B, C. The TEM images also exhibited the nanosheet morphology, with clear lattice fringes of 0.33 and 0.29 nm. The EDX-elemental mapping images show that the elemental composition of the Co/MoSe_2_/PPy carbon nanofibers is carbon (C), nitrogen (N), cobalt (Co), molybdenum (Mo), and selenium (Se) (Fig. 2G–L). The distribution elements of Mo, Co, N, and Se are identical to those of carbon, consistent with the uniform Co/MoSe_2_/PPy sheets implanted on CNFs. Energy-dispersive X-ray spectroscopy (EDX) spectra of the Co/MoSe_2_/PPy NF and CNF samples are also presented in Fig. S1, which further confirmed the coexistence of C, N, Co, Mo, and Se in the obtained samples. The SEM images in Fig. 2D demonstrate that Co/MoSe_2_/PPy@CNF exhibits a relatively porous and rough surface with diameters ranging from 850 nm to 1 μm. The TEM images (Fig. 2E, F) show that the layered Co/MoSe_2_/PPy nanosheets with 0.33 nm spaced crystal plane (002) were extensively grown in carbon-based spun networks. Thus, the TEM results of the Co/MoSe_2_/PPy@NF and Co/MoSe_2_/PPy@CNF samples are consistent with the SEM-EDX mapping results. Additionally, the SEM images of PAN, MoSe_2_, MoSe_2_@PPy, and Co/MoSe_2_ NFs and their CNFs are presented in the Supporting Information (Fig. S2 and S3). An obvious change in surface morphology was observed in 2D-TMD-based NFs and their CNFs. The electrospun PAN NFs (Fig. S2A) are uniform in size and exhibit a smooth surface and a cross-linked or network morphology. Figure S2B-C shows that MoSe_2_ nanosheets were deposited locally on the NF surfaces. As shown in Fig. S2-D, the MoSe_2_ nanosheets were embedded in the fibers synthesized from the Co/MoSe_2_ nanolayer structures.

**Fig. 3:**
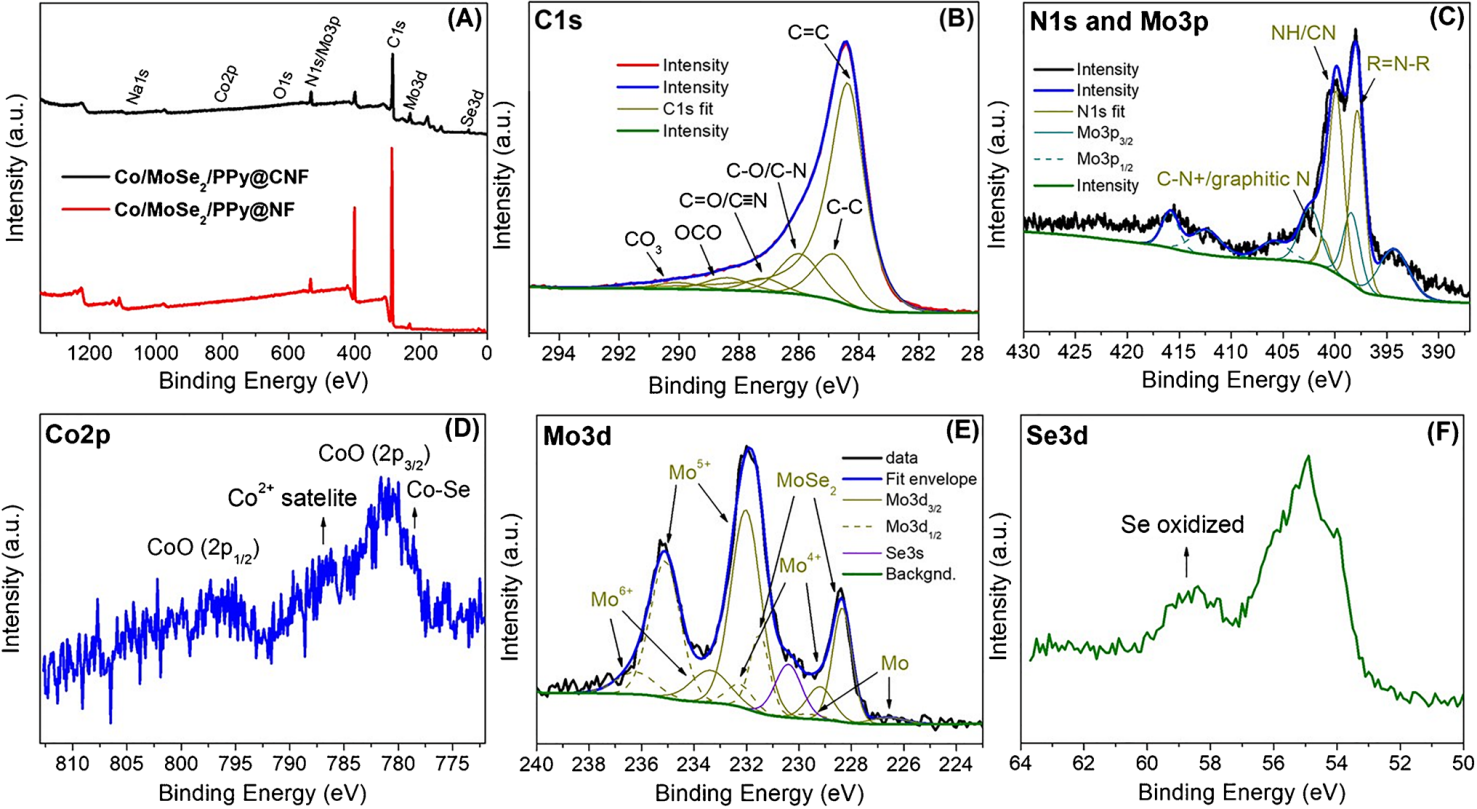
XPS spectra of Co/MoSe_2_/PPy@CNFs: **A** survey spectrum in comparison with Co/MoSe_2_/PPy@NF, **B** C1s spectrum, **C** N1s and Mo3p region, **D** Co2p spectrum, **E** Mo3d spectrum, and **F** Se3d spectrum

XPS measurements were performed to investigate the chemical composition and oxidation states of the atoms in Co/MoSe_2_/PPy@CNFs (Fig. 3). The XPS spectrum shows signals in the C1s, O1s, Mo3d, Se3d, Co2p, Na1s, and N1s and Mo3p regions (Fig. 3A). The high-resolution spectra and further deconvolution revealed an overlap of the N1s signal and Mo3p signal (Fig. 3C) as well as overlap of the Mo3d and Se3s signals (Fig. 3E). The peak at 399.7 eV in the N1s spectra can be assigned to the C≡N group of PAN and 397.8 eV to a nitrogen atom within the pyrrole core as a building block of PPy [39]. In addition, after deconvolution, a small peak at 401.1 eV can be assigned to the formation of the C-N^+^ form as a result of doping by Co^2+^ or eventually graphitic N [40]. A complex Mo3d spectrum can result from multiple forms of molybdenum present in Mo^6+^, Mo^5+^, Mo^4+^, Mo, and MoSe_2_, in which all species show two peaks for Mo3d_1/2_ and Mo3d_3/2_. Molybdenum present in higher oxidation states can be attributed to surface oxidation, which occurs upon exposure to air or during the carbonization process [40, 41]. The Se3d spectrum showed a peak corresponding to Mo-Se bonds (Fig. 3F) and generally involves two peaks at 54.1 eV (Se3d_3/2_) and 54.9 eV (Se3d_1/2_) [42, 43]. Additionally, the Se–O peak at 58.7 eV can be assigned to SeO_2_ formed from the exposure of Co/MoSe_2_/PPy@CNFs to air [44, 45]. A small peak at 58.3 eV corresponding to the Se-O bond was visible in the Se3d spectra of MoSe_2_@CNF (Fig. S4 and Table S1) and MoSe_2_/PPy@CNF (Fig. S4). A small intensity XPS signal was visible in the Co2p spectra, complicating the spectrum interpretation (Fig. 3D). The predominant signal can be assigned to Co2p_1/2_ and Co2p_3/2_ of CoO species [46], which can result from surface oxidation during the carbonization process. A small signal visible at ~778.5 eV could be attributed to the Co-Se bond [47, 48]. The C1s spectrum (Fig. 3B) contains multiple peaks corresponding to C=C, C-C, C-O/C-N, and C=O/C≡N bonds at 284.4, 284.8, 286.0, and 287.2 eV, respectively, with an additional shoulder, which can be due to the presence of O-C=O species (288.4 eV) [49, 50] and CO_3_ species (290 eV) [51]. The chemical composition and oxidation states of all atoms are listed in the Supplementary Information (Table S1).

### Electrochemical results

**Fig. 4:**
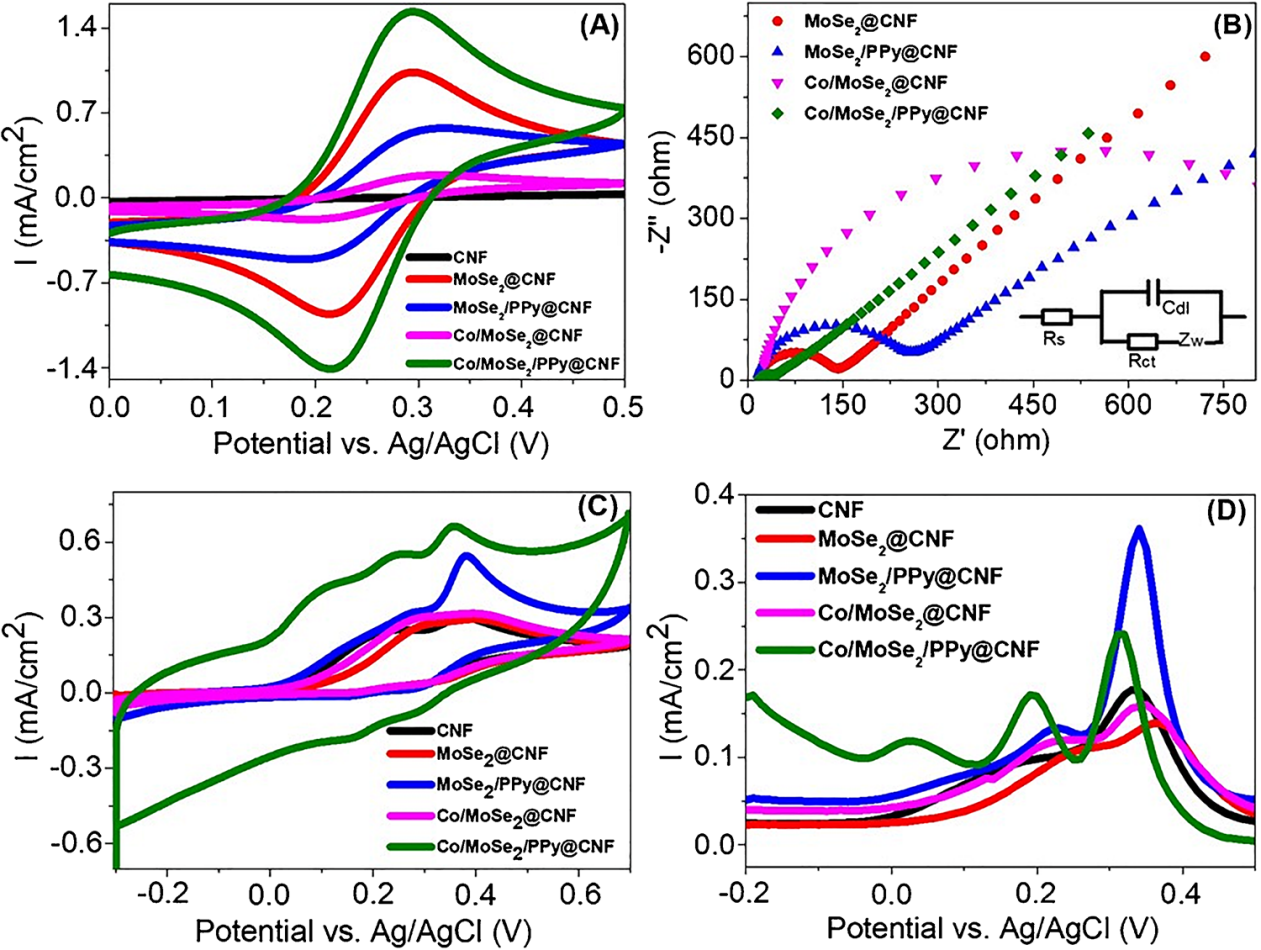
**A** CVs of various electrodes in 5 mM [Fe(CN_6_)]^3−^/^4−^ containing 0.1 M KCl. **B** EIS curves and corresponding equivalent circuit (inset) of various electrodes in 5 mM [Fe(CN_6_)]^3−^/^4−^ containing 0.1 M KCl. *R*_ct_ is the charge transfer resistance, *R*_s_ is the resistance of the electrolyte solution, *C*_dl_ is the double-layer capacitance, and *Z*_w_ is the Warburg impedance. **C** CVs and **D** DPVs of various electrodes in 0.1 M PBS solution containing 1.48 mM AA, 0.19 mM DA, and 0.74 mM UA

To determine the electrochemical behaviors of the 2D-TMD@CNF-coated electrodes, CV was applied in 5.0 mM [Fe (CN_6_)]^3−/4−^ containing 0.1 M KCl for a potential range from −0.2 to +0.5 V at a scan rate of 50 mV/s. As clearly seen in Fig. 4A, all electrodes demonstrated well-separated redox peaks associated with the ferri/ferrocyanide couple. The Co/MoSe_2_/PPy@CNF (1.58 mA/cm^2^/−1.45 mA/cm^2^)-modified GCE exhibited higher anodic/cathodic peak currents compared to those of CNF (0.05 mA/cm^2^/−0.04 mA/cm^2^), MoSe_2_@CNF (1.08 mA/cm^2^/−1.01 mA/cm^2^), MoSe_2_/PPy@CNF (0.62 mA/cm^2^/−0.54 mA/cm^2^), and Co/MoSe_2_@CNF (0.22 mA/cm^2^/−0.23 μA). The improved electrochemical properties of GCE-Co/MoSe_2_/PPy@CNF could be attributed to the larger electroactive surface area and higher conductivity due to the incorporation of PPy and Co into the 2D-nanostructured MoSe_2_ nanolayers. In addition, electroactive surface areas of GCE-CNF, GCE-MoSe_2_@CNF, GCE-MoSe_2_/PPy@CNF, GCE-Co/MoSe_2_@CNF, and GCE-Co/MoSe_2_/PPy@CNF were determined from cyclic voltammograms using the Randles-Sevcik equation [52].

$$I_\text{p}=\left(2.69\times10^5\right){A\;D}^{1/2}n^{3/2}v^{1/2}C$$

where *A* is the electrode surface area (cm^2^), *D* is the diffusion coefficient (7.6 × 10^−6^ cm^2^ s^−1^), *n* is the number of electrons involved in the process (*n* = 1), *C* is the concentration of [Fe(CN)6]^3−/4−^ (5 × 10^−6^ M), *ν* is the scan rate (0.05 V s^−1^), and *I*_p_ is the intensity of peak current (A). The electroactive surface area values of GCE-CNF, GCE-MoSe_2_@CNF, GCE-MoSe_2_/PPy@CNF, GCE-Co/MoSe_2_@CNF, and GCE-Co/MoSe_2_/PPy@CNF were calculated to be 0.0171, 0.4105, 0.2352, 0.0863, and 0.599 cm^2^, respectively. The findings suggest that the utilization of GCE-Co/MoSe_2_/PPy@CNF results in a significantly expanded electroactive surface area. Thus, Co, PPy, and MoSe_2_ emerge as beneficial materials for enhancing the electron transfer efficiency and sensitivity of electrode systems. In addition, EIS was performed to define the electronic transfer characteristics of all GCE-CNF electrodes in 0.1 M KCl containing 5 mM [Fe (CN)_6_]^3−/4−^ with a frequency range of 100 mHz–100 kHz and an applied potential of 0.25 V. Figure 4B represents the Nyquist plots of CNF, MoSe_2_@CNF, MoSe_2_/PPy@CNF, Co/MoSe_2_@CNF, and Co/MoSe_2_/PPy@CNF, and the inset figure shows the corresponding equivalent circuit. Nyquist curves in EIS, consisting of semicircular parts at high frequencies and straight lines at low frequencies, are related to the electrochemical kinetic control step, in which the semicircular radius indicates the charge transfer resistance. The semicircle area is related to the charge transfer (*R*_ct_) limited operation, and the diameter of this semicircle could be used to determine the interfacial electron transport property of the electrode [53]. The *R*_ct_ values were estimated to be 135 Ω (GCE-MoSe_2_@CNF), 250 Ω (GCE-MoSe_2_/PPy@CNF), 1200 Ω (Co/MoSe_2_@CNF), and 30 Ω (Co/MoSe_2_/PPy@CNF). The *R*_ct_ value of the Co/MoSe_2_/PPy@CNF-modified electrode clearly exhibited a relatively better electron transfer rate than that of CNF, MoSe_2_@CNF, MoSe_2_/PPy@CNF, and Co/MoSe_2_@CNF, which corresponds well with the CV results. The relatively better charge transfer feature of GCE-Co/MoSe_2_/PPy@CNF can be attributed to the increased surface area and electrical conductivity of the interface material. The electrocatalytic activities of GCE electrodes modified with various 2D-TMD-based CNF materials were obtained by CV and DPV in 0.1 M PBS containing 1.48 mM AA, 0.19 mM DA, and 0.74 mM UA. Figure 4C shows the voltammograms, which were performed between −0.3 and 0.7 V at a scan rate of 50 mV/s. There was no obvious peak separation for AA, DA, and UA on the CNF-, MoSe_2_@CNF-, MoSe_2_/PPy@CNF-, and Co/MoSe_2_@CNF-modified GCEs. On the other hand, GCE-Co/MoSe_2_/PPy@CNF possessed the best electrocatalytic activity with well-separated peak potentials toward AA, DA, and UA. The higher electrocatalytic activity of the Co/MoSe_2_/PPy@CNF-modified electrode could be attributed to the incorporation of PPy and Co, which leads to more catalytic active sites on the MoSe_2_ nanolayers and improved conductivity. The electrocatalytic activities of the 2D-TMD@CNF-modified GCE electrodes were also investigated through DPV measurements, which is generally a much more sensitive analytical technique than CV. From Fig. 4D, only two oxidation peaks were acquired for GCE-CNF (DA, 0.18 V; UA, 0.34 V), GCE-MoSe_2_@CNF (DA, 0.26 V; UA, 0.36 V), and GCE-Co/MoSe_2_@CNF (DA, 0.23 V; UA, 0.34 V), which indicated that the AA and DA overlapped. For the MoSe_2_/PPy@CNF-modified electrode, three oxidation peaks were observed at 0.05 V, 0.23 V, and 0.34 V, defining the oxidation potentials of AA, DA, and UA, respectively. Although the UA oxidation peak current on the GCE-MoSe_2_/PPy@CNF sufficiently provides high detection sensitivity, the AA and DA oxidation peak currents are too low to generate high detection sensitivities. Compared with other modified electrodes, GCE-Co/MoSe_2_/PPy@CNF demonstrated the best electrocatalytic activities and well-separated oxidation potentials at 0.03 V, 0.19 V, and 0.32 V with high peak currents for AA, DA, and UA, respectively. Notably, the enhanced peak currents and large peak potential separations on the GCE-Co/MoSe_2_/PPy@CNF electrode provided an efficient sensing platform for the simultaneous detection of AA, DA, and UA.

**Fig. 5:**
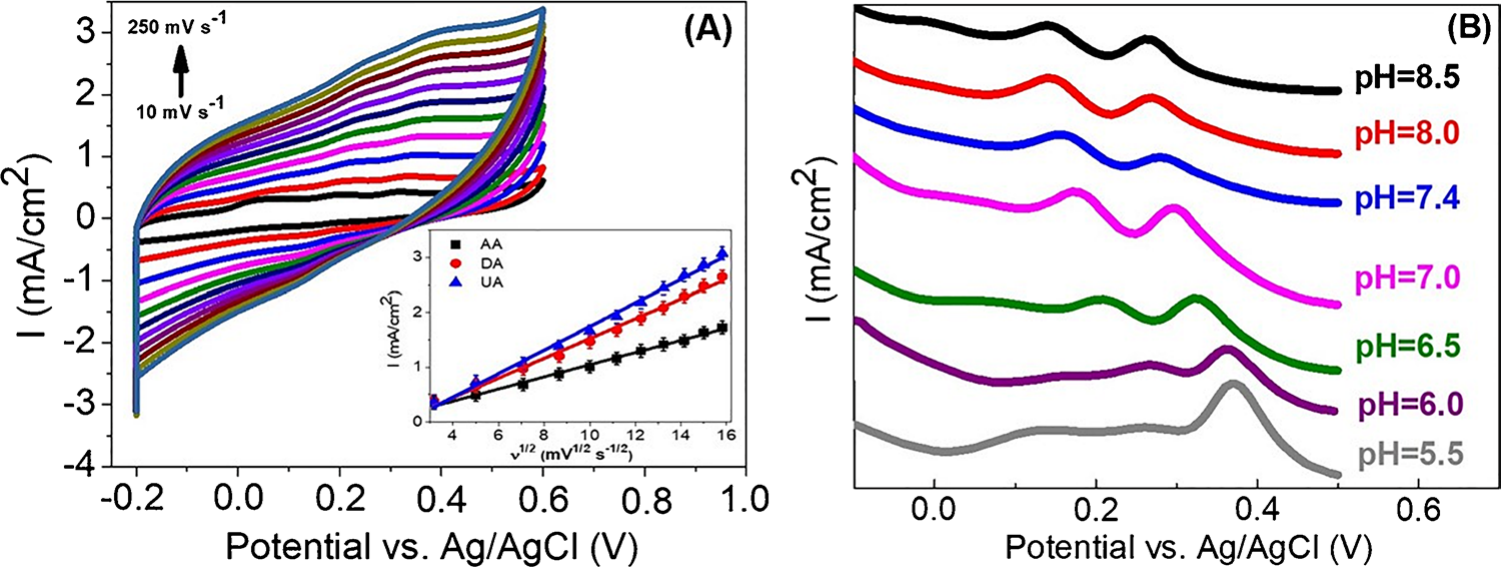
**A** CV curves of GCE-Co/MoSe_2_/PPy@CNF in 0.1 M PBS (pH 7.0) containing 2.14 mM AA, 0.19- mM DA, and 0.74 mM UA at different scan rates from 10 to 250 mV/s. Inset figure shows anodic peak currents vs. square roots of scan rates. **B** DPVs of 2.14 mM AA, 0.19 mM DA, and 0.74 mM UA at GCE-Co/MoSe_2_/PPy@CNF at various pH values (from 5.5 to 8.5). The scan rate was 50 mV s^−1^

To determine the reaction kinetics and the effect of different scan rates (10–250 mV/s) on the electrocatalytic oxidations of AA (2.14 mM), DA (0.19 mM), and UA (0.74 mM) on the Co/MoSe_2_/PPy@CNF-modified GCE electrode, CV was employed in 0.1 M PBS (pH 7.0) and is shown in Fig. 5A. The anodic and cathodic oxidation peak currents for AA, DA, and UA increased linearly with the square root of the scan rates (Fig. 5A inset). These relationships were defined by the regression equations of the anodic for AA, DA, and UA as follows:

$$AA:{I}_{\textrm{pa}}\left(\upmu \textrm{A}\right)=\left(111.0963\pm 2.5621\right)\ {v}^{1/2}{\left(\textrm{V}/\textrm{s}\right)}^{1/2}-0.0631\pm 0.0288;\;{R}^2=0.9953$$

$$DA:{I}_{\textrm{pa}}\left(\upmu \textrm{A}\right)=\left(180.2670\pm 4.8743\right)\ {v}^{1/2}{\left(\textrm{V}/\textrm{s}\right)}^{1/2}-0.2776\pm 0.0547;\;{R}^2=0.9935$$

$$UA:{I}_{\textrm{pa}}\left(\upmu \textrm{A}\right)=\left(215.2017\pm 5.5286\right)\ {v}^{1/2}\ {\left(\textrm{V}/\textrm{s}\right)}^{1/2}-0.4068\pm 0.0619;\kern0.5em {R}^2=0.9937$$

These results disclose that the oxidation of AA, DA, and UA on GCE-Co/MoSe_2_/PPy@CNF was a diffusion-controlled process [54]. Figure 5B shows the DPV curves of the Co/MoSe_2_/PPy@CNF-modified electrode in buffer solutions with different pH values (from 5.5 to 8.5) containing 2.14 mM AA, 0.19 mM DA, and 0.74 mM UA. Compared with other pH values, pH 7.0 appeared to be the optimal pH value for AA, DA, and UA detection, showing the best electrocatalytic activity and well-separated oxidation potential.

**Fig. 6:**
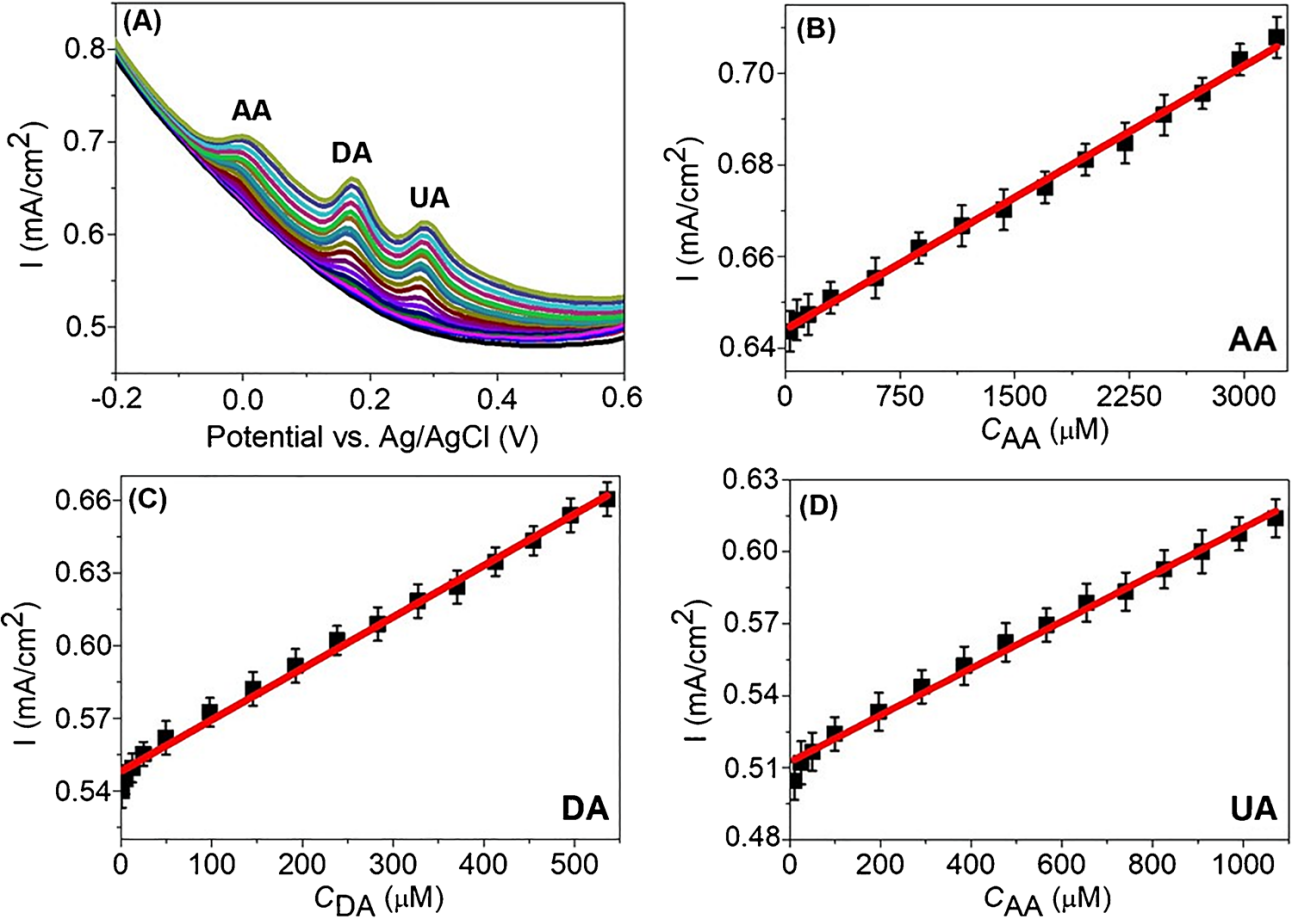
**A** DPVs of GCE-Co/MoSe_2_/PPy@CNF in 0.1 M PBS (pH 7.0) containing various concentrations of AA, DA, and UA. Calibration plots of **B** AA, **C** DA, and **D** UA obtained from their anodic peak currents versus concentrations

Simultaneous detection of AA, DA, and UA on GCE-Co/MoSe_2_/PPy@CNF was carried out through DPV. Figure 6A displays the DPV curves for increasing concentrations of AA, DA, and UA in 0.1 M PBS (pH 7.0). Clearly, the oxidation peak locations of AA (0.03 V), DA (0.19 V), and UA (0.32 V) maintain peak-to-peak potential separation as their concentrations are gradually increased. Figure 6B–D represents the corresponding calibration curves acquired from DPVs for linear concentration ranges of 30–3212 μM for AA, 1.2–536 μM for DA, and 10–1071 μM for UA. Furthermore, the calibration graphs showed the following linear regression equations and determination coefficients:

$$AA:\varDelta {I}_{\textrm{pa}}\left(\textrm{mA}\right)=\left(0.019\pm 3.099\times {10}^{-4}\right)\ {C}_{\textrm{AA}}\left(\textrm{mM}\right)+0.644\pm 5.885\times {10}^{-4};\;{R}^2=0.996$$

$$DA:{\varDelta I}_{pa}\left(\textrm{mA}\right)=\left(0.213\pm 4.512\times {10}^{-3}\right)\ {C}_{\textrm{DA}}\left(\textrm{mM}\right)+0.548\pm 1.271\times {10}^{-3};{R}^2=0.993$$

$$UA:{\varDelta I}_{\textrm{pa}}\left(\textrm{mA}\right)=\left(0.098\pm 2.401\times {10}^{-3}\right){C}_{\textrm{UA}}\left(\textrm{mM}\right)+0.512\pm 1.441\times {10}^{-3};{R}^2=0.992$$

According to the slope (*S*) of the regression equations and standard deviation (Sb) of the mean values of 10 DPVs of the blank solution, LODs were calculated to be 6.32, 0.45, and 0.81 μM for AA, DA, and UA, respectively, by using the 3Sb/S equation [55]. Compared with other electrodes modified with various TMD- and CNF-based materials, the Co/MoSe_2_/PPy@CNF-modified GCE exhibits good and comparable analytical performance for the simultaneous determination of AA, DA, and UA (Table 1).

**Table 1:** Comparison of Co/MoSe_2_/PPy@CNF for AA, DA, and UA detection with other similar electrodes

Electrode	Linear range (μM)			LOD (μM)			Ref
	AA	DA	UA	AA	DA	UA	
MoS_2_/PEDOT	20–140	1–80	2–25	5.83	0.52	0.95	[56]
rGO/PPy-Pt	0.8–2.1	0.03–1.4	0.1–0.35	0.12	0.071	0.16	[8]
Ti-C-Tx/GCE	100−1000	0.5–50	0.5–4; 100–1500	4.64	0.06	0.075	[54]
AuNPs@MoS_2_-NSs	2–300	5–200	20–400	3	1	5	[57]
3D MoS_2_-PANI/rGO	50–5.0 × 10^3^	5–500	1–500	22.70	0.70	0.36	[58]
NCNF	50~3000	1~10 10~200	5~200	50	0.5	1	[59]
CB-CNT/PI	1000–24,000	3–300	5–500	154	1.86	3	[60]
Fe_3_O_4_/Co_3_O_4_/mC@g-C_3_N_4_/GCE	500–8000	1–70	5–100	12.55	0.21	0.18	[61]
Co/MoSe_2_/PPy@CNF	30–3212	1.2–536	10–1071	6.32	0.45	0.81	This work

The mechanism for the simultaneous and selective determination of AA, DA, and UA on the Co/MoSe_2_/PPy@CNF can be described as follows: Co-doping into MoSe_2_ in the presence of conducting polymer increases the catalytic active centers and also leads to the formation of anionic Se vacancies, which facilitate the oxidation reactions of AA, DA, and UA [29, 62]. For DA, the electrocatalytic reaction may involve the conversion of catechol group to o-quinone [63]. DA becomes negatively charged by losing protons during this reaction. The anionic defects on the Co/MoSe_2_/PPy@CNF may combine these protons, thus increasing the positive charges on the electrode. The positively charged surface of the electrode is able to interact with the analytes to increase the amount of adsorption. For UA, the catalytic mechanism is related to the oxidation of the bridging double bond into -OH followed by dehydration [64]. In the case of AA, the hydroxyl groups on the furan ring can be easily oxidized to carbonyl groups and AA is converted to dehydroascorbic acid [65]. Along with their oxidation mechanism, AA, DA, and UA with different structures have different interaction mechanisms on the Co/MoSe_2_/PPy@CNF electrode. For example, AA, DA, and UA may have different π-π interactions with PPy in the hybrid structure [54]. The above discussions clearly indicate that different interactions result in separation of the oxidation peak potentials and provide the possibility for simultaneous detection of the mixture of AA, DA, and UA.

**Fig. 7:**
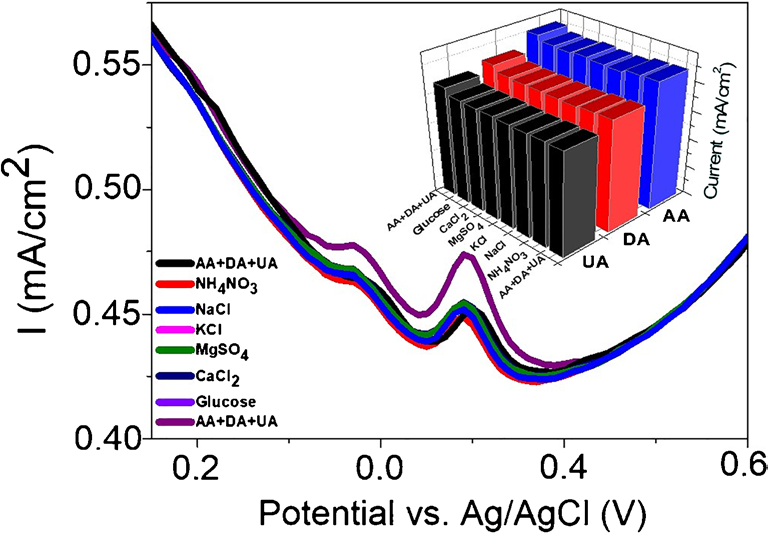
DPVs of GCE-Co/MoSe_2_/PPy@CNF in 0.1-M PBS containing 1.0 mM interfering substances in the presence of 0.58 mM AA, 0.03 mM DA, and 0.19 mM UA

There are diverse substances that coexist with AA, DA, and UA in real samples, which can disturb the electrochemical signals of the analytes. Therefore, DPV measurements were applied to determine the selectivity of the developed sensor by adding these substances at higher concentrations into the mixed solution of AA, DA, and UA. Figure 7 shows that 1.0 mM NaNO_3_, NaCl, KCl, MgSO_4_, NH_4_Cl, glucose, and citric acid did not influence the analytical signals of 0.58 mM AA, 0.03 mM DA, and 0.19 mM UA, indicating the selectivity of the sensor. Furthermore, reproducibility is a significant parameter in the development of electrochemical sensors. Therefore, DPV was also applied for five freshly different electrodes arranged under the same circumstances. These electrodes show 6.5, 3.0, and 2.8% of the relative standard deviation (RSD) for AA, DA, and UA, respectively, indicating convincing reproducibility. The stability of the Co/MoSe_2_/PPy@CNF electrode was also studied by storing the electrode in a refrigerator for a period of 10 days. DPV measurements showed that the electrode retained the initial concentrations of AA, DA, and UA by 87.2%, 85.6%, and 92.0%, respectively. Such a decrease is attributed to the dissolution of some part of the materials from the electrode surface.

### Determination of DA, AA, and UA in a human urine sample

The feasibility of the suggested Co/MoSe_2_/PPy@CNF-modified GCE electrode is verified through practical application for the determination of AA, DA, and UA in human serum samples by DPV via a standard addition method. Before the measurement, a fresh urine sample was diluted to 100 times with 0.1 M PBS solution (pH 7) and spiked with known concentration of 30 μM of AA, DA, and UA, respectively. From the obtained DPVs, recoveries for AA, DA, and UA were calculated as between 94.0 and 105.5% and RSDs were between 0.9 and 7.4% by measurements (Table S2). These analysis results indicated that the proposed GCE-modified electrode can be applied successfully into the simultaneous detection of AA, DA, and UA in real samples.

## Conclusion

In this contribution, 2D-TMD@CNF nanocomposites were prepared successfully as electrode modifiers and used to produce an electrochemical sensor for the simultaneous detection of AA, DA, and UA. Owing to the excellent electrocatalytic activity of metal-doped 2D-TMD/polymer-based nanocomposites, along with the high surface area and porosity properties of CNF structures, an electrode constructed Co/MoSe_2_/PPy@CNF was able to individually and simultaneously detect AA, DA, and UA. Additionally, the Co/MoSe_2_/PPy@CNF-modified electrode provided separated oxidation peaks for AA, DA, and UA with better current responses. The limit of detection aligned with established scientific literature, affirming the capability of the fabricated electrode to accurately quantify all three analytes. Furthermore, successful detection of these analytes within authentic urine samples was achieved. The results clearly showed that the electrode constructed with new types of 2D-TMD@CNF-based hybrid materials can be evaluated for real-time prevention and diagnosis of diseases as electrochemical sensing platforms that simultaneously detect AA, DA, and UA.

### **Supplementary information**

ESM 1 (DOCX 2221 kb)

## Data Availability

Data will be made available on request.
