# Supplementary material for: Electrospun cobalt-doped 2D-MoSe2/polypyrrole hybrid-based carbon nanofibers as electrochemical sensing platforms
Source: Mikrochim Acta. 2024 Jan 4;191(1):75. doi: 10.1007/s00604-023-06078-2 (PMC10764547; doi:10.1007/s00604-023-06078-2)
Supplement: Supplementary file 1 — (DOCX 2221 kb) [file 604_2023_6078_MOESM1_ESM.docx]

**Supporting information**

**Electrospun cobalt-doped 2D-MoSe_2_/polypyrrole hybrid based carbon nanofibers as electrochemical sensing platforms**

**Gamze Celik Cogal^1,2*^, Sadik Cogal^1,3^, Peter Machata^1^, Aysegul Uygun Oksuz^2^, Maria Omastová^1^**

*^1^Polymer institute, Slovak Academy of Sciences, Dubravska cesta 9,
84541 Bratislava, Slovakia*

*^2^Suleyman Demirel University, Faculty of Arts and Science, Department of Chemistry, 32000, Isparta, Türkiye3^c^Burdur Mehmet Akif Ersoy University, Faculty of Arts and Science, Department of Chemistry, 15030, Burdur, Türkiye*

[*gamze.celik-cogal@savba.sk](mailto:*gamze.celik-cogal@savba.sk); [gamzecelikcogal@gmail.com](mailto:gamzecelikcogal@gmail.com)


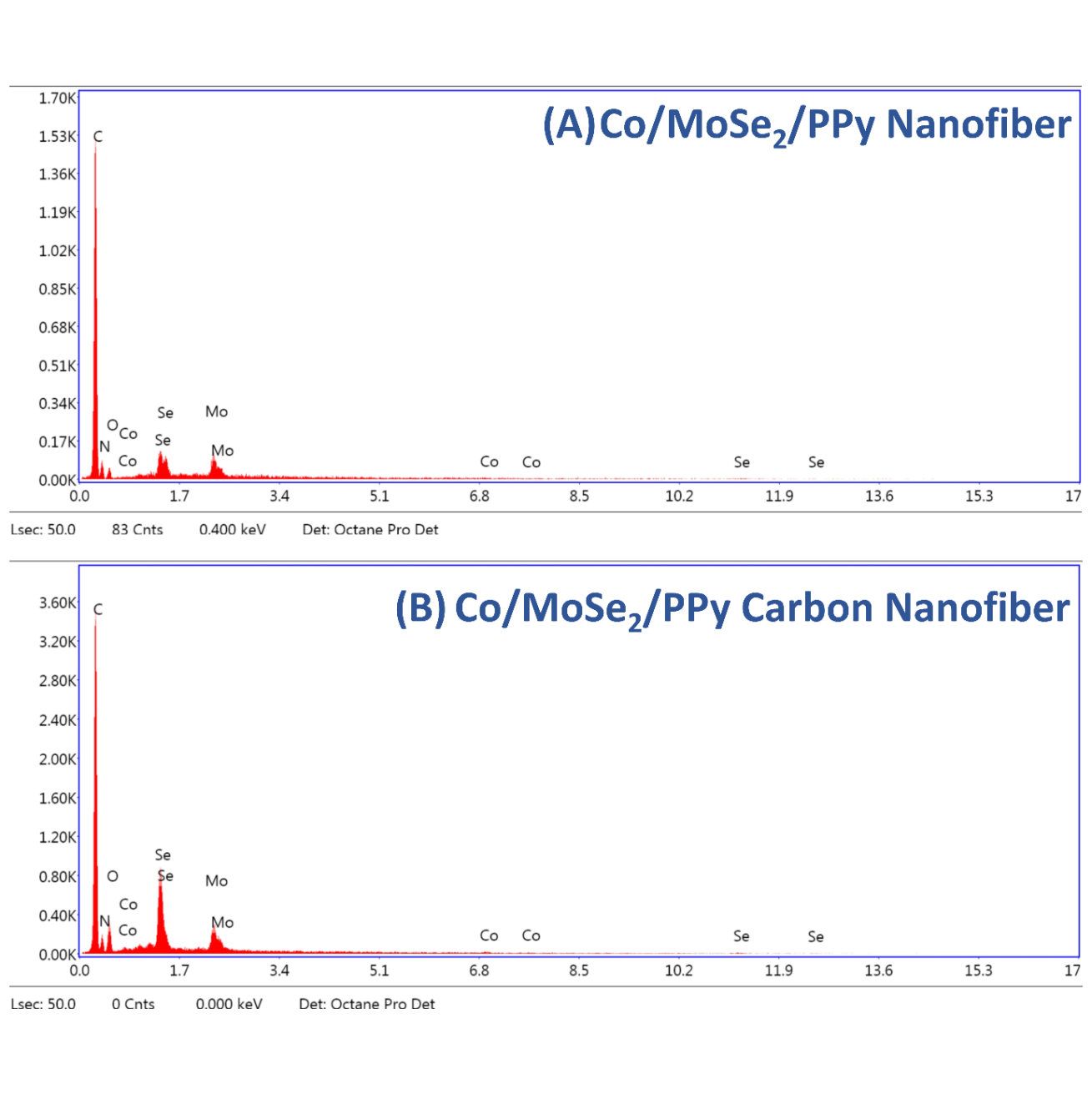


**Fig. S1.** EDX analysis of Co/MoSe_2_/PPy; (A) as-spun nanofibers and (B) carbon nanofibers.


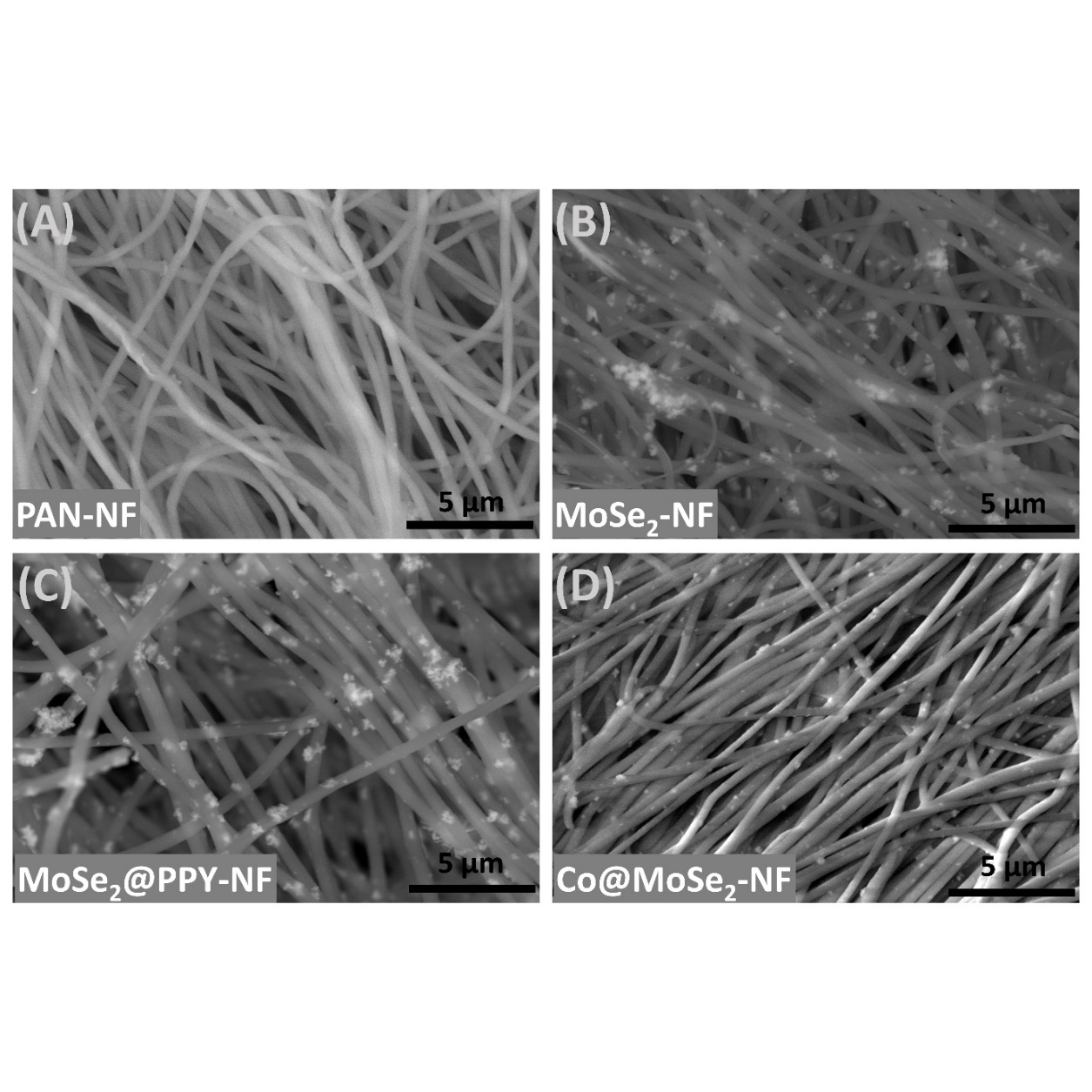


**Fig. S2.** SEM images of as-spun (A) PAN, (B) MoSe_2_, (C) MoSe_2_/PPy, and (D) Co/MoSe_2_ nanofibers.


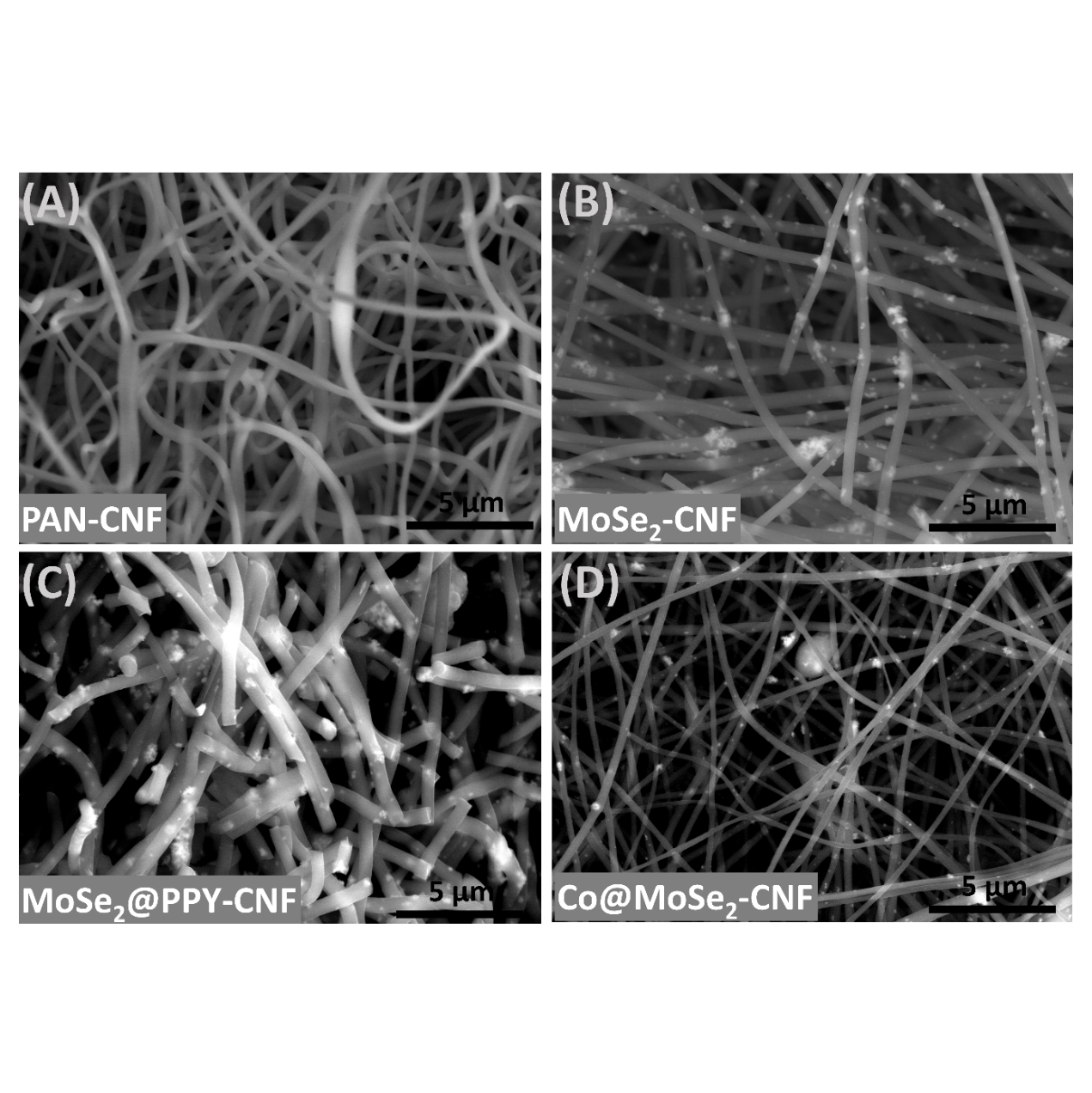


**Fig. S3.** SEM images of (A) PAN, (B) MoSe_2_, (C) MoSe_2_/PPy, and (D) Co/MoSe_2_ carbon nanofibers.


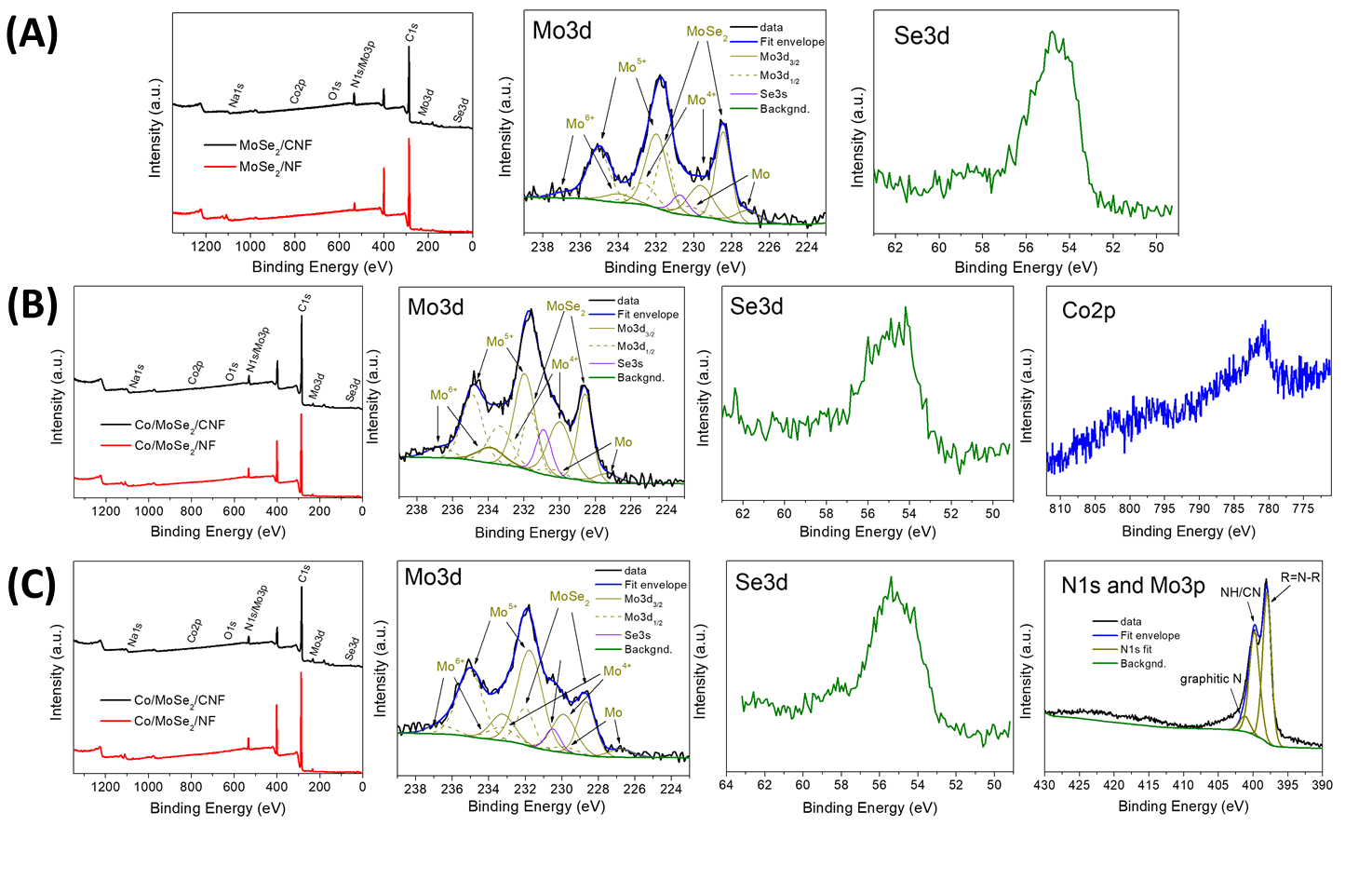


**Fig. S4.** XPS survey and high resolution spectra of (A) MoSe_2_@CNFs, (B) Co/MoSe_2_@CNFs, and (C) MoSe_2_/PPy@CNFs.

**Table S1.** Apparent surface chemical composition of all samples as determined by XPS.

| sample |  | Surface chemical composition (at.%) | | | | | |
| --- | --- | --- | --- | --- | --- | --- | --- |
|  | C1s  C=C/ C-C/  C-N (C-O)/ C=O(C≡N)/ OCO/CO_3_/π-π | | O1s | Se3d | N1s  (R=N-R)/ C≡N(NH)/ graphitic | Mo3d  Mo^5+^/Mo^6+^/MoSe_2_/  Mo^4+^/Mo | Co2p/  Na1s |
| MoSe_2_@CNF | 76.7  38.8/21.4/11.6/1.5/2.7/0.7/<0.1 | | 7.0 | 0.6 | 15.3  (7.8/6.3/1.2) | 0.3  0.1/<0.1/0.1//<0.1//<0.1/ | -/0.1 |
| Co/MoSe_2_@CNF | 77.7  44.7/14.8/11.0/2.6/2.5/0.8/1.3 | | 4.0 | 0.2 | 21.6  (9.3/7.2/1.1) | 0.3  0.1/<0.1/0.1/<0.1//<0.1/ | <0.1/- |
| MoSe_2_/PPy@CNF | 77.8  40.4/19.4/10.8/2.9/3.2/0.9/1.5 | | 4.7 | 0.6 | 16.2  (8.4/6.8/1.0) | 0.61  0.3/<0.1/0.1/0.1/<0.1 | -/0.10 |
| Co/MoSe2/PPy@CNF | 78.9  52.3/10.1/8.8/2.7/3.0/1.5/<0.1 | | 7.6 | 1.3  - | 10.6  (4.7/5.2/0.6) | 1.3  0.7/0.1/0.2/0.2/<0.1 | 0.1/  0.2 |

**Table S2.** DPV analysis of spiked AA, DA and UA in diluted human urine samples using Co/MoSe_2_/PPy@CNF modified electrode as an electrochemical sensor (n=3).

| Sample | Analyte | Added (μM) | Found  (μM) | Recovery | RSD |
| --- | --- | --- | --- | --- | --- |
|  | AA  DA  UA | -  -  - | -  -  45 | -  -  - | -  -  3.1 |
|  | AA | 250 | 244 | 97.5 | 7.4 |
|  | DA | 25 | 23.5 | 94 | 1.8 |
|  | UA | 50 | 93 | 96 | 1.5 |
| Urine |  |  |  |  |  |
|  | AA | 500 | 510 | 102 | 4.2 |
|  | DA | 50 | 48 | 96 | 2.3 |
|  | UA | 100 | 143.5 | 98.8 | 0.9 |
|  |  |  |  |  |  |
|  | AA | 750 | 745 | 99.3 | 3.5 |
|  | DA | 75 | 72.5 | 96.7 | 1.4 |
|  | UA | 150 | 201.5 | 104.3 | 1.2 |
|  |  |  |  |  |  |
|  |  |  |  |  |  |
